# Multi-Probing Opportunistic Routing in Buffer-Constrained Wireless Sensor Networks

**DOI:** 10.3390/s26082295

**Published:** 2026-04-08

**Authors:** Nannan Sun, Shouxin Cao, Xiaoyuan Liu, Yue Gao, Yang Xu, Jia Liu

**Affiliations:** 1 China Satellite Network Application Co., Ltd., Beijing 100160, China; nnasun_88@126.com; 2 School of Computer Science and Technology, Xidian University, Xi’an 710126, China; shouxin.cao@stu.xidian.edu.cn (S.C.); ygao@xidian.edu.cn (Y.G.);; 3 Beijing Petroleum Machinery Co., Ltd., Beijing 102206, China; liuxiaoydr@cnpc.com.cn

**Keywords:** wireless sensor networks, multiple probing strategy, opportunistic routing, limited buffer capacity, performance evaluation

## Abstract

Wireless sensor networks (WSNs) are fundamental building blocks of modern ubiquitous sensing systems. In many practical WSN deployments, sensing devices are tightly constrained in buffer capacity, while device mobility leads to topology decentralization. These characteristics pose significant challenges for reliable and timely data delivery across WSNs. In this paper, we propose a general multi-probing opportunistic routing strategy tailored for buffer-constrained WSNs, aiming to enhance transmission opportunity utilization under realistic sensing device limitations. With the help of Queueing Theory and Markov Chain Theory, we capture the sophisticated queueing processes for the buffer space of sensors, which enables the limiting distribution of the buffer occupation state to be determined. On this basis, we develop a theoretical performance modeling framework to evaluate the fundamental performance metrics of the WSN with the multi-probing opportunistic routing, including the per-flow throughput and the expected end-to-end delay. The validity of the performance modeling framework is verified by network simulations. Moreover, extensive numerical results demonstrate the network performance behaviors comprehensively and reveal some insightful findings that can serve as important guidelines for the configuration and operation of WSNs.

## 1. Introduction

The wireless sensor network (WSN) is a key enabling technology for modern ubiquitous sensing systems, which essentially consist of interconnected smart sensing devices capable of monitoring physical environments, transmitting sensed data, and performing distributed computing without human intervention [1,2]. Recent years have witnessed a rapid proliferation of WSN deployments, and it is expected that the number of devices with sensing capabilities worldwide will reach tens of billions. As an emerging technology paradigm, WSNs have been increasingly adopted in a wide range of large-scale industrial applications, including intelligent manufacturing, smart logistics, and critical infrastructure monitoring in oil–gas systems [3].

In many large-scale industrial and ubiquitous computing applications, sensing devices are typically deployed in a decentralized manner and operate under strict energy and hardware constraints. Due to device mobility, intermittent activation, and limited transmission power, wireless connectivity among sensors becomes highly dynamic and opportunistic, resulting in decentralized network topologies and intermittent links. As a consequence, reliable end-to-end paths (routes) between source and destination devices generally do not exist. In this context, opportunistic routing, which treats the shared wireless medium as an opportunity rather than a limitation and adopts the store–carry–forward strategy, has become a promising solution for achieving end-to-end data delivery in decentralized WSN scenarios [4].

The fundamental idea underlying opportunistic routing is to exploit the broadcast nature of the wireless medium to mitigate the drawbacks of unreliable wireless transmission. More specifically, instead of pre-selecting a specified path between a source–destination pair, opportunistic routing allows the source to send its packets to an intermediate device, called a relay, if they are within the transmit range; the relay stores and carries the packets, and can forward the packets later when encountering the destination. Mhemed et al. [5] conducted a comprehensive survey on opportunistic routing in underwater WSNs, with a particular focus on void-avoiding mechanisms, by analyzing the causes of communication voids and reviewing representative protocols along with their strengths and limitations. Farooq et al. [6] proposed an opportunistic routing in asynchronous WSNs by adopting a software-defined networking architecture to centrally control candidate selection and prioritize forwarding nodes for balancing sender waiting time, duplicate packets, and energy consumption. Based on the analytical hierarchy process and fuzzy inference, Wang et al. [7] introduced a multi-criteria decision framework to address the forwarder selection problem for opportunistic routing in asynchronous duty-cycled WSNs. Xian et al. [8] designed a delay-aware opportunistic routing in maritime search and rescue WSNs, where link connectivity prediction and expected packet advancement were jointly exploited to improve data delivery timeliness and energy efficiency. Considering the uncertain topology and frequent routing voids in underwater WSNs, Zhu et al. [9] developed a cluster-based routing scheme that combines void prediction and link connectivity estimation with an opportunistic forwarding strategy. To address performance degradation in non-uniformly distributed and densely deployed WSNs, Lata et al. [10] proposed an opportunistic routing by introducing a local density-aware forwarder selection strategy based on fuzzy c-means clustering. More recently, Wang et al. [11] presented a game-theoretic opportunistic routing framework for underwater WSNs by modeling relay selection as a bargaining process to achieve energy-efficient and void-resilient data forwarding.

### 1.1. Motivations

It is worth noting that, to enable the store–carry–forward operation of opportunistic routing, intermediate devices must be equipped with buffers. However, due to the lightweight architecture and severe resource constraints of sensing devices, the available buffer space is typically very limited, and buffer occupancy has a direct and significant impact on the achievable quality of service (QoS). As a result, routing designs that ignore buffer constraints may lead to overly optimistic performance assessments and are often unsuitable for practical WSN deployments. Moreover, in most existing opportunistic routing schemes, when a node gains access to the wireless channel, it probes only a single neighboring node for potential transmission if the destination is not within its communication range. Although such a single-probing strategy is simple to implement, it can severely waste precious transmission opportunities in highly dynamic WSNs. Specifically, when a transmitter attempts to forward a packet to a randomly probed relay, the relay’s buffer may already be full; conversely, when the transmitter itself serves as a relay, it may have no buffered packet destined for the probed neighbor. In both cases, the transmission opportunity is lost despite successful channel access, resulting in inefficient use of scarce wireless resources. These observations motivate the need for a more efficient opportunistic routing strategy that jointly considers buffer availability and the utilization of transmission opportunities.

### 1.2. Contributions

To cope with the aforementioned limitations, in this paper, we investigate opportunistic routing in decentralized dynamic WSN under practical buffer space constraints. We develop a general multi-probing opportunistic routing strategy that explicitly accounts for buffer availability, aiming to improve the utilization of transmission opportunities in highly dynamic and resource-constrained scenarios. To characterize the resulting system behaviors, we establish a rigorous analytical framework based on queueing theory and Markov chain theory, which captures the stochastic buffer dynamics of sensing devices and enables the limiting distribution of buffer occupancy to be derived. Building upon this framework, we further develop theoretical performance models to evaluate the fundamental network performance, including per-flow throughput and expected end-to-end delay, and validate the analytical results through extensive network simulations.

The main contributions of this paper are summarized as follows:

- We develop a general multi-probing opportunistic routing scheme for decentralized WSNs under realistic buffer space constraints. The proposed design jointly integrates a structured buffer space management mechanism, a cell-partitioned contention-based channel access strategy, and a two-hop relaying routing algorithm with sequential multi-probing capability. By allowing a transmitter to probe up to ρ neighboring devices upon acquiring channel access, the scheme significantly mitigates transmission opportunity waste caused by buffer unavailability and dynamic neighborhood variations. The overall design enables more efficient utilization of scarce wireless transmission opportunities and improves end-to-end data delivery performance in highly dynamic WSNs.
- We establish a rigorous analytical framework to characterize the stochastic buffer evolution induced by the proposed routing scheme. By leveraging queueing theory and Markov chain modeling, we derive the steady-state distribution of buffer occupancy and tractable expressions for fundamental performance metrics, including per-flow throughput and expected end-to-end delay. The proposed analysis provides a systematic understanding of how mobility, buffer constraint, and probing depth jointly shape network performance.
- We conduct extensive network simulations to validate the accuracy of the theoretical performance modeling and to comprehensively investigate the performance behaviors of the proposed multi-probing opportunistic routing scheme. The numerical results provide valuable insights into the impacts of system parameters on network performance, offering practical guidelines for the configuration and operation of buffer-constrained WSNs.

### 1.3. Paper Organization

The remainder of this paper is organized as follows. In Section 2, we introduce the related work. Section 3 provides the network model involved in this study. We propose the multi-probing opportunistic routing scheme in Section 4 and conduct the network performance evaluation in Section 5. Section 6 presents the simulation results, followed by the conclusion in Section 7.

## 2. Related Work

Routing has long been recognized as a fundamental research problem in wireless sensor networks, as it directly affects data delivery reliability, latency, and energy efficiency, which are critical to enabling sensor-driven embedded and ubiquitous computing applications. Rani et al. [12] investigated routing optimization in industrial WSNs by enhancing the OLSR protocol with fault-tolerant clustering and control interval management, demonstrating improved QoS performance for smart grid applications. Almesaeed et al. [13] focused on routing design for mobile WSNs under diverse application requirements and developed a dynamic directional routing protocol to enhance network lifetime and packet delivery performance. To prolong the network lifetime of software-defined WSNs, an energy-aware routing scheme was proposed in [14], which leverages centralized control to balance energy consumption and reduce control overhead. SureshKumar et al. [15] designed an energy-efficient and trust-aware routing model for wireless sensor networks by integrating intelligent optimization techniques for cluster head selection and secure path establishment. Shah et al. [16] studied cooperative routing design for underwater WSNs and developed a neighbor-based energy-efficient protocol that exploits broadcast transmission to enhance network lifetime and data delivery performance. To improve energy efficiency and network stability in WSNs, Wang et al. [17] proposed a hybrid intelligent clustering and routing framework by combining fuzzy logic with quantum annealing. Zhang et al. [18] investigated adaptive clustering routing in WSNs and employed swarm intelligence-based optimization to balance energy consumption and prolong network lifetime. Li et al. [19] proposed a trust-aware hierarchical routing framework for WSNs that integrates multi-level trust assessment with energy awareness to enhance security and network robustness. These studies collectively indicate that routing design in WSNs has been extensively explored from multiple perspectives, underscoring its fundamental role in sensor-enabled ubiquitous computing systems.

While extensive efforts have been devoted to optimizing routing efficiency in relatively stable WSN settings, emerging sensor-enabled ubiquitous computing scenarios increasingly exhibit dynamic topology changes, intermittent connectivity, and heterogeneous link conditions, rendering conventional deterministic routing strategies insufficient. To cope with such uncertainties, the opportunistic routing paradigm has attracted increasing research attention, aiming to exploit real-time link availability and node mobility to improve data delivery reliability. Considering dynamic link conditions in agricultural WSNs, Wu et al. [20] proposed an energy-efficient opportunistic routing scheme that leverages algebraic connectivity for forwarder selection and residual-energy-aware backoff to reduce redundant transmissions. Inspired by swarm intelligence, Chaurasia et al. [21] developed an optimized opportunistic routing protocol that employs a dragonfly-based metaheuristic to jointly optimize forwarder selection and routing paths based on residual energy and distance. In [22], a trust-aware load-balanced opportunistic routing scheme was proposed for asynchronous duty-cycled WSNs, where energy-, topology-, and trust-related metrics are jointly incorporated to guide forwarding decisions. Zhang et al. [23] addressed routing attacks in underwater WSNs by designing a clustering-based intrusion detection scheme for opportunistic routing, which improves data transmission reliability in adversarial environments. Considering the limited communication range and directional constraints in underwater optical WSNs, Yan et al. [24] designed an energy-balanced opportunistic routing protocol that incorporates void handling and energy-aware relay selection. Despite these advances, a systematic understanding of how buffer constraints interact with transmission opportunity utilization in opportunistic routing is still lacking.

Another important research line related to this work concerns the performance evaluation of WSNs, which seeks to analytically characterize key performance metrics under different routing and network configurations. Zroug et al. [25] developed a formal modeling and analysis framework for WSN protocols based on hierarchical timed colored Petri nets, demonstrating its effectiveness for performance evaluation beyond simulation-based approaches. Khalifeh et al. [26] investigated the use of WSNs for smart city monitoring applications and conducted experimental performance evaluations in real outdoor environments. Behera et al. [27] provided an overview of clustered routing designs in WSNs and comparatively evaluated their performance characteristics under different cluster head selection mechanisms. A learning-based routing framework for WSNs was investigated in [28], where clustering and routing decisions are optimized using intelligent algorithms, and the network performance was analyzed in terms of lifetime, energy consumption, and packet delivery ratio. Rahman et al. [29] conducted a simulation-based performance comparison of routing protocols for WSNs using multiple QoS metrics. Xu et al. [30] evaluated WSN deployment strategies from a performance-centric perspective and demonstrated how performance-aware modeling can significantly improve overall network behavior. Collectively, these works underscore the critical role of performance evaluation in understanding and improving WSN behavior under different routing and network configurations.

## 3. Network Model and Performance Metrics

This section introduces the network model adopted in this study and performance metrics used for evaluation.

### 3.1. Network Model

**Figure 1:**
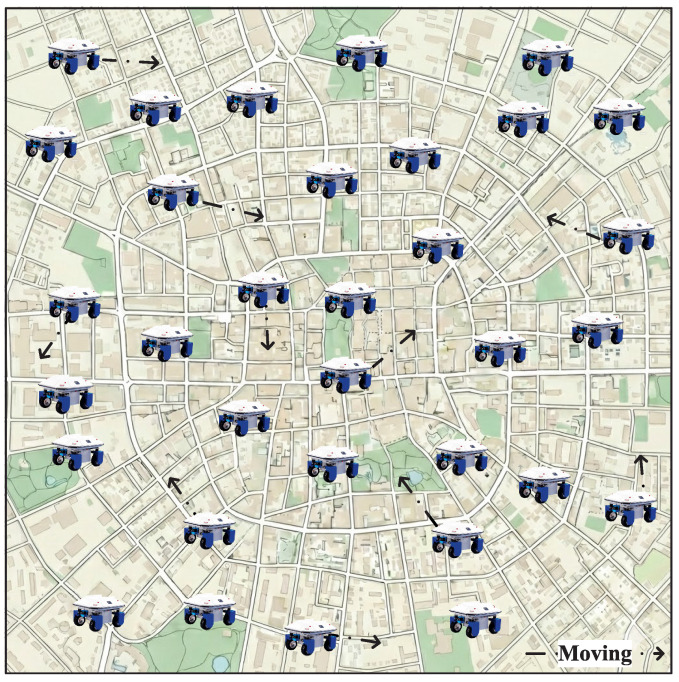
Network model.

We consider a general decentralized WSN as illustrated in Figure 1, composed of *N* (N≥3) sensing devices (sensor nodes) deployed over a torus-shaped region. Each sensor node is equipped with a limited communication range and moves randomly over the network area. The node mobility follows a time-slotted uniform mobility model [31], under which the spatial position of each sensor forms a stationary and ergodic stochastic process with a uniform steady-state distribution. The mobility processes of different sensors are assumed to be independent and identically distributed. The torus topology is adopted to eliminate boundary effects and ensure spatial homogeneity. It can be interpreted as an approximation of large-scale dense deployments, such as environmental monitoring or mobile sensing systems over wide geographic areas, where edge effects are negligible, and nodes experience statistically similar interaction patterns regardless of their locations.

The network supports *N* unicast data flows, where each sensor simultaneously serves as the source of one flow and the destination of another, resulting in a permutation traffic pattern, which has been widely adopted in the literature for decentralized mobile networks, e.g., [31,32]. Time is divided into slots, and the transmission capacity of each sensor within one slot is normalized to one packet. Exogenous packet arrivals at each sensor are modeled as independent Bernoulli processes with mean rate λ, such that a new packet is generated at the end of a time slot with probability λ. Moreover, to capture practical resource limitations, each sensor node is assumed to be equipped with a finite buffer space that can store at most *B* packets.

### 3.2. Performance Metrics

To quantify the performance of the buffer-constrained decentralized WSN under consideration, we focus on two fundamental metrics, namely the per-flow throughput and the expected end-to-end delay, which are defined below.

*Per-Flow Throughput*: The throughput of an individual traffic flow is denoted by Γ, and is defined as the long-term average rate at which exogenous packets generated by a source sensor are successfully delivered to its corresponding destination, measured in packets per time slot.

*Expected End-to-End Delay*: Let Θ represent the end-to-end (E2E) delay experienced by a packet, defined as the total number of time slots elapsed from the moment the packet enters the source node’s buffer until it is received by its destination. The average E2E delay is then denoted by E{Θ}.

## 4. Multi-Probing Opportunistic Routing Scheme

This section introduces the multi-probing opportunistic routing scheme for the considered WSN. Owing to the limited communication range of sensing devices, direct transmission of exogenous packets from a source to its destination is generally infeasible. In addition, sensor node mobility and decentralized operation make it difficult to establish and maintain stable multi-hop routes in such networks.

To enable reliable E2E data delivery under these conditions, the proposed scheme adopts a two-hop relaying framework, which exploits device mobility to create transmission opportunities and benefit from multi-user diversity. Within this framework, a multi-probing mechanism is incorporated to improve the utilization of channel access opportunities, together with a lightweight buffer management strategy that supports store–carry–forward operations required by two-hop relaying. In addition, a contention-based channel access control mechanism is incorporated to regulate medium access among neighboring devices. Together, these components constitute a unified routing scheme tailored for the buffer-constrained and dynamically evolving WSN.

### 4.1. Buffer Space Management

**Figure 2:**
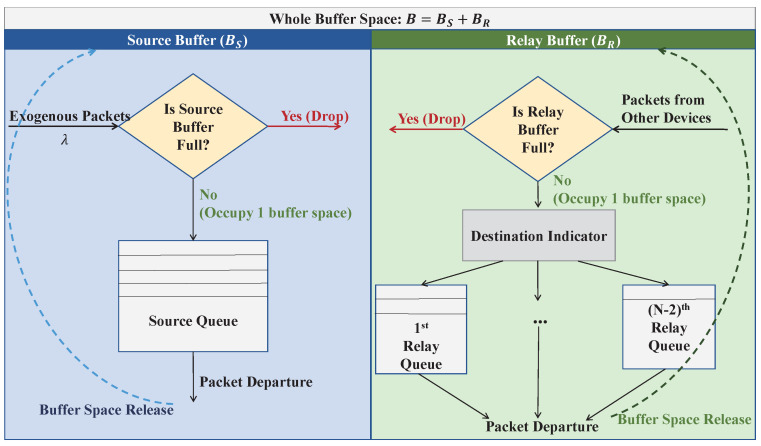
Buffer space management.

The buffer space management mechanism adopted in this work is illustrated in Figure 2. Each sensor node is equipped with a finite buffer of size *B* packets, reflecting the inherent resource constraints of decentralized WSNs. To accommodate the opportunistic routing operation, the whole buffer space is logically partitioned into two components: a source buffer of size $B_{S}$ and a relay buffer of size $B_{R}$, such that $B_{S}+B_{R}=B$.

The source buffer is exclusively reserved for storing packets generated by the node itself (i.e., the exogenous packets), whereas the relay buffer is used to temporarily store packets belonging to other traffic flows during the relaying process. To distinguish different relayed flows, the relay buffer is further organized into N−2 virtual relay queues, each corresponding to one traffic flow. All queues operate under a first-in-first-out (FIFO) service discipline.

Packet admission to the buffer is subject to capacity constraints. Specifically, when an exogenous packet arrives at a device whose source buffer is full, the packet is immediately discarded. Similarly, when a packet received from another device arrives while the relay buffer is fully occupied, it is dropped upon arrival. Once a packet departs from the source queue or any relay queue due to a successful transmission, the corresponding buffer space is released and becomes available for subsequent arrivals.

By adjusting the partitioning of the buffer space between $B_{S}$ and $B_{R}$, the proposed framework allows flexible control over the tradeoff between accommodating self-generated traffic and supporting relay operations, which in turn has a direct impact on the overall network performance. This buffer organization provides a structured basis for the queueing analysis of the proposed routing scheme, which will be shown in Section 5.

### 4.2. Channel Access Control

**Figure 3:**
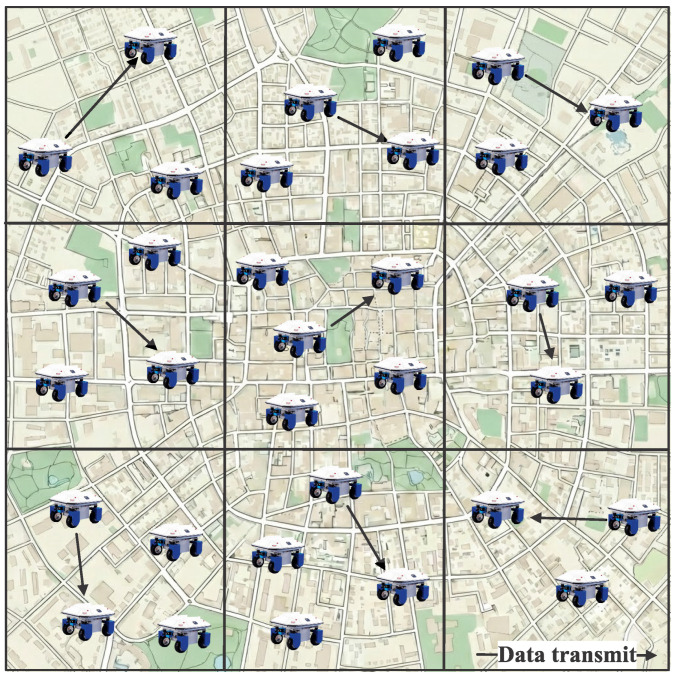
Cell-partitioned structure-based media access control.

To support the multi-probing opportunistic routing scheme, a structured channel access control (MAC) mechanism is employed to regulate concurrent transmissions among sensor nodes. The design follows a cell-partitioned framework that has proven effective in decentralized mobile wireless networks [31], while providing a tractable abstraction for routing and performance analysis. Specifically, the network area is evenly divided into a set of non-overlapping square cells, forming an M×M tessellation, as illustrated in Figure 3. This spatial partitioning enables localized channel access decisions and limits contention to nodes residing in the same cell. During each time slot, at most one transmitter–receiver pair is permitted to communicate within a cell, and packet transmissions across different cells are not allowed.

To mitigate wireless interference caused by simultaneous transmissions in nearby regions, orthogonal frequency channels are assigned to adjacent cells, ensuring that concurrent communications do not interfere with each other. Within each cell, channel access is determined through a distributed contention procedure. At the beginning of each time slot, all nodes currently located in the same cell participate in a contention process inspired by the IEEE 802.11 distributed coordination function (DCF) [33]. Each node independently selects a back-off timer uniformly at random from the interval (0,WD], where WD denotes the contention window size. The node whose timer expires first gains access to the channel and initiates transmission in that slot.

This cell-based access control mechanism provides a simple yet effective means to coordinate medium access in a fully decentralized manner, while enabling analytical characterization of transmission opportunities under node mobility. As a result, the channel access process in each cell can be viewed as generating at most one transmission opportunity per time slot. It is worth noting that, under this MAC mechanism, packet collisions and signaling interference are implicitly avoided, as at most one node can successfully transmit within each cell in a given time slot. Moreover, the sequential multi-probing procedure is performed only after a node acquires the channel, and thus does not introduce additional contention among nodes. The probing handshake is assumed to be lightweight and reliable, and its signaling overhead is abstracted in the analytical model.

### 4.3. Routing Algorithm

Based on the buffer space management and channel access control mechanisms described in the previous subsections, we now detail the routing operations performed by a sensor node once it successfully acquires the wireless channel. Due to the decentralized nature of the WSN and the limited transmission range of sensor nodes, routing decisions are made locally at each transmitting node based solely on instantaneous neighborhood information. Consider a generic transmitting node that has obtained channel access in a given time slot. The routing decision starts by examining the relative location of its intended destination. If the destination node is located in the same cell, direct packet delivery becomes feasible. In this case, the node follows the *source-to-destination (S–D) operation* described in Procedure 1. Specifically, the transmitting node sends the head-of-line (HoL) packet from its source queue to the destination, provided that the source queue is non-empty; otherwise, the node remains idle for the current slot.

When the destination is not within the transmission range, i.e., it resides in a different cell, direct delivery is not possible, and the transmitting node resorts to relay-assisted forwarding. To this end, a two-hop relaying strategy is adopted, under which the transmitting node probabilistically selects between two alternative forwarding modes. With probability α, the node initiates a *source-to-relay (S–R) operation*, and with probability 1−α, it attempts a *relay-to-destination (R–D) operation*. This randomized selection allows the WSN to balance packet injection into relay buffers and packet draining toward final destinations.
**Procedure 1** Source-to-destination (S-D) operation  1:**if **S has packets in its source queue **then**  2:    S transmits the head-of-line (HoL) packet in its source queue to D;  3:    S removes the HoL packet from its source queue;  4:    S moves ahead the remaining packets in its source queue;  5:**else**  6:    S remains idle;  7:**end if**

If the S–R mode is selected, the transmitting node executes the procedure detailed in Procedure 2. In this operation, the transmitting node seeks to offload a packet from its source queue to a neighboring relay node located in the same cell. Since relay buffer availability is limited and unknown a priori, a multi-probing strategy is employed. The transmitter sequentially probes candidate relay nodes, each selected uniformly at random from the set of nodes in the same cell. Upon probing a candidate relay, a lightweight handshake is performed to check whether sufficient relay buffer space is available. If the relay buffer is not full, the HoL packet is transmitted, stored in the corresponding relay queue at the relay node, and removed from the source queue. The probing process then terminates immediately. If the relay buffer is full, the transmitter continues probing another candidate relay until either a feasible relay is found or the probing limit ρ is reached. If no eligible relay is identified within the first ρ−1 probing attempts, the transmitter performs a final transmission attempt in the ρ-th probing round. In this case, the packet is transmitted regardless of the relay buffer state. If buffer space is available, the relay stores the packet; otherwise, the packet is dropped. In both cases, the packet is removed from the source queue, ensuring that the source queue does not block indefinitely. Note that this design does not affect the system stability, which is fundamentally governed by the queue dynamics under finite buffer capacities. Since both the source and relay buffers are bounded, the queue lengths remain inherently stable. The rationale behind this mechanism is to prioritize newly arriving packets over stale ones when the source buffer is congested, thereby improving information freshness without compromising stability.

On the other hand, if the R–D mode is selected, the transmitting node follows the procedure specified in Procedure 3. In this case, the transmitting node attempts to deliver packets stored in its relay buffer to their respective destinations. Similar to the S–R case, a multi-probing procedure is applied. The transmitter randomly selects a neighboring node in the same cell and checks whether it carries relay packets destined for the selected receiver. If such packets exist, the HoL packet of the corresponding relay queue is transmitted, removed from the relay buffer, and the released buffer space becomes immediately available for future relay traffic. If no matching relay packet is found, the transmitter continues probing until the probing limit ρ is reached, after which the transmission opportunity is relinquished.

Overall, the multi-probing strategy plays a critical role in improving the utilization of scarce transmission opportunities by reducing idle slots caused by buffer overflow or empty relay queues. Due to the symmetry among sensor nodes and traffic flows, the above routing behavior is representative for all source–destination pairs. In the subsequent analysis, we focus on a tagged flow with source S and destination D, where S executes the proposed multi-probing two-hop relaying opportunistic routing scheme (MP-2HROR) upon each successful channel access, as formally summarized in Algorithm 1.
**Procedure 2** Source-to-relay (S-R) operation  1:**if **S has packets in its source queue **then**  2:    i←1;  3:    **while** i<ρ **do**  4:        With equal probability, S randomly selects a device (say $R_{i}$) out of the devices in its same cell;  5:        S initiates a handshake with $R_{i}$ to check whether the relay buffer of $R_{i}$ is full or not;  6:        **if** The relay buffer of $R_{i}$ is not full **then**  7:            S transmits the HoL packet in its source queue to $R_{i}$;  8:            $R_{i}$ allocates a relay buffer space for the relay queue corresponding to this packet and pushes the packet to the end of the relay queue;  9:            S removes the HoL packet from its source queue;10:            S moves ahead the remaining packets in its source queue;11:            i←ρ+1;12:        **else**13:            i←i+1;14:        **end if**15:    **end while**16:    **if** i=ρ **then**17:        With equal probability, S randomly selects a device (say $R_{\rho }$) out of the devices in its same cell;18:        S transmits the HoL packet in its source queue to $R_{\rho }$;19:        **if** The relay buffer of $R_{\rho }$ is not full **then**20:            $R_{\rho }$ allocates a relay buffer space for the relay queue corresponding to this packet and pushes the packet to the end of the relay queue;21:        **else**22:            $R_{\rho }$ drops this packet.23:        **end if**24:        S removes the HoL packet from its source queue;25:        S moves ahead the remaining packets in its source queue;26:    **end if**27:**else**28:    S remains idle.29:**end if**

**Procedure 3** Relay-to-destination (R-D) operation
  1:**if **S has packets in its relay queues **then**  2:    i←1;  3:    **while** i≤ρ **do**  4:        With equal probability, S randomly selects a device (say $V_{i}$) out of the devices in its same cell;  5:        S checks whether it carries packets destined to $V_{i}$;  6:        **if** S carries packets destined to the receiver **then**  7:            S transmits the HoL packet in its corresponding relay queue to $V_{i}$;  8:            S removes the HoL packet from this relay queue;  9:            S moves ahead the remaining packets in this relay queue;10:            This relay queue releases one buffer space to the relay buffer of S;11:            i←ρ+1;12:        **else**13:            i←i+1;14:        **end if**15:    **end while**16:
**else**
17:    S remains idle.18:
**end if**



**Algorithm 1** Multi-Probing Two-hop Relaying Opportunistic Routing Algorithm (MP-2HROR)
  1:S checks whether D is in its same cell;  2:**if **D is within the same cell of S **then**  3:    S executes Procedure 1;  4:
**else**
  5:    With probability α, S selects to do S-R operation; With probability 1−α, S selects to do R-D operation;  6:    **if** S selects S-R operation **then**  7:        S executes Procedure 2;  8:    **else**  9:        S executes Procedure 3;10:    **end if**11:
**end if**



To facilitate the theoretical performance analysis of MP-2HROR, we characterize the probabilities associated with different forwarding modes. Let $p_{sd}$, $p_{sr}$, and $p_{rd}$ denote the probabilities that a tagged sensor node S performs an S–D, S–R, and R–D operation, respectively, upon obtaining a transmission opportunity in a time slot. Under the adopted cell-partitioned MAC mechanism, whether a specific routing operation is executed depends jointly on the instantaneous spatial configuration of nodes and the outcome of channel contention within the cell. In particular, the occurrence of an S–D (resp. S–R or R–D) operation in a given slot requires the simultaneous satisfaction of several conditions: (i) the relative location condition, i.e., whether the destination D is located in the same cell as S (for S–D) or not (for S–R/R–D); (ii) the neighborhood configuration, namely that exactly *i* out of the remaining N−2 nodes are present in the same cell as S, while the other N−2−i nodes reside in different cells; and (iii) successful channel access by S within that cell under the contention-based MAC mechanism. By combining the above spatial and contention-related events, the probabilities $p_{sd}$, $p_{sr}$, and $p_{rd}$ can be expressed as follows:

(1) psd=∑i=0N−2N−2i1M2i+11−1M2N−2−i·1i+2=∑i=0N−2N−1i+11M2i+11−1M2N−2−i·1i+2−∑i=0N−3N−2i+11M2i+11−1M2N−2−i·1i+2=M2N1−1−1M2N−1−1M2N−1−M2−1N−11−1−1M2N−1+1−1M2N−1=M2N−M2−1N−1+M2−1N(N−1)1−1M2N−1,

(2) psr=α∑i=1N−2N−2i1M2i1−1M2N−1−i·1i+1=αM2−1N−1−M2N−11−1M2N−1−1M2N−1,

(3) prd=(1−α)∑i=1N−2N−2i1M2i1−1M2N−1−i·1i+1=(1−α)M2−1N−1−M2N−11−1M2N−1−1M2N−1.

## 5. Theoretical Performance Evaluation

This section develops a theoretical framework to quantify the performance of the proposed MP-2HROR scheme. We begin by characterizing the steady-state occupancy distributions of both the source and relay buffers. Based on these distributions, closed-form expressions for the per-flow throughput and the expected E2E delay are derived.

### 5.1. Source Buffer Occupancy State Distribution

Under the routing scheme described in the previous section, packet arrivals and departures at the source queue follow a slot-based stochastic evolution. Specifically, in each time slot, an exogenous packet arrives at the source queue with probability λ. Meanwhile, a packet at the head of the source queue is successfully served whenever S executes either an S–D or S–R operation. Since these events occur with probabilities $p_{sd}$ and $p_{sr}$, respectively, the effective service probability of the source queue is given by $\mu_{S}=p_{sd}+p_{sr}$. Therefore, the source queue can be modeled as a discrete-time Bernoulli arrival/Bernoulli service queue with finite capacity $B_{S}$.

Let $\phi_{k}$ denote the steady-state probability that *k* packets occupy the source buffer, where $k\in \{0,1,\ldots ,B_{S}\}$. Denote the steady-state distribution vector of source buffer occupancy by $\Phi_{\lim}=\left[\phi_{0},\phi_{1},\ldots ,\phi_{B_{S}}\right]$. Following the standard analysis of finite-capacity Bernoulli queues [34], $\phi_{k}$ can be determined as

$$\phi_{k}=\left\{\begin{array}{ccc} \frac{1}{1-\lambda }Z_{\mathrm{norm}}^{-1}, & \; & k=0\\ \frac{1}{1-\lambda }\frac{\tau^{k}}{1-\mu_{S}}Z_{\mathrm{norm}}^{-1}, & \; & 1\leq k\leq B_{S} \end{array}\right.$$

where

(4) $$\tau =\frac{\lambda (1-\mu_{S})}{\mu_{S}\left(1-\lambda \right)},$$

and $Z_{\mathrm{norm}}$ is the normalization constant.

By enforcing the normalization condition $\sum_{k=0}^{B_{S}}\phi_{k}=1$, then we have

(5) $$\phi_{k}=\left\{\begin{array}{cc} \frac{\mu_{S}-\lambda }{\mu_{S}-\lambda \tau^{B_{S}}}, & k=0,\\ \frac{\mu_{S}-\lambda }{\mu_{S}-\lambda \tau^{B_{S}}}\frac{1}{1-\mu_{S}}\tau^{k}, & 1\leq k\leq B_{S}. \end{array}\right.$$

### 5.2. Relay Buffer Occupancy State Distribution

We next characterize the stochastic evolution of the relay buffer at a tagged node S. Let $R\left(t\right)\in \left\{0,1,\ldots ,B_{R}\right\}$ denote the number of packets stored in the relay buffer at the beginning of time slot *t*. The process {R(t)} captures the aggregate behavior of all relay queues maintained at node S. Due to the operation rules of the MP-2HROR scheme, in any given time slot node S can either receive a relay packet (through an S–R operation initiated by another node) or forward one of its stored relay packets to a destination (through an R–D operation), but these two events cannot occur simultaneously. Consequently, the relay buffer occupancy evolves as a one-dimensional birth-death process with finite capacity $B_{R}$. More specifically, when the current state is *w*, the relay buffer may experience one of the following transitions in the next slot:

- *No change* (w→w): neither a new relay packet arrives nor an existing relay packet departs;
- *Increment* (w→w+1), for $0\leq w<B_{R}-1$: another node successfully delivers a packet to S, and the packet is admitted into the relay buffer;
- *Decrement* (w→w−1), for $1\leq w\leq B_{R}$: node S successfully forwards an HoL packet from one of its relay queues to its corresponding destination.

Let $T_{w,w^{\prime }}$ denote the one-step transition probability from state *w* to state $w^{\prime }$, and we derive $T_{w,w^{\prime }}$ as follows.

(1) Upward Transition Probability $T_{w,w+1}$

An increment of the relay buffer occurs when node S is selected as a relay and successfully receives a packet from another node. Note that the departure rate $\lambda_{p}$ from a generic source queue is $\lambda_{p}=\mu_{S}\left(1-\phi_{0}\right)$, where $\phi_{0}$ is the probability that the source buffer is empty. Given that a packet departs from a source queue, it is forwarded to a relay with probability $\frac{p_{sr}}{\mu_{S}}$. When node S serves as a relay candidate, there are N−2 potential transmitting nodes (excluding itself and its own destination). Each transmitting node selects a relay uniformly at random among the N−2 candidates. Due to the multi-probing mechanism, S may be selected in different probing rounds. Let $p_{f}$ denote the steady-state probability that a relay buffer is full and define $\xi =\frac{N-3}{N-2}p_{f}$, which represents the probability that a randomly chosen relay candidate (excluding S) is unavailable because its buffer is full.

If S is selected successfully in the first probing round, the probability is $\frac{1}{N-2}\left(1-p_{f}\right)$. If selection occurs in the *t*-th probing round (t<ρ), the probability is $\xi^{t-1}\frac{1-p_{f}}{N-2}$, since the previous t−1 candidates are unavailable. For the ρ-th probing round, the selection probability is $\xi^{\rho -1}\frac{1}{N-2}$. Summing over all possible probing rounds and accounting for the aggregate arrival attempts from N−2 nodes, the upward transition probability is calculated as

(6) $$\begin{array}{cc} T_{w,w+1} & =\left(N-2\right)\lambda_{p}\cdot \frac{p_{sr}}{\mu_{S}}\cdot \sum_{t=1}^{\rho }\left[\xi^{t-1}\frac{1-p_{f}}{N-2}\right]+\left(N-2\right)\lambda_{p}\cdot \frac{p_{sr}}{\mu_{S}}\cdot \xi^{\rho -1}\frac{1}{N-2}\\ \; & =p_{sr}\left(1-\phi_{0}\right)\left[\frac{\left(1-p_{f}\right)\left(1-\xi^{\rho -1}\right)}{1-\xi }+\xi^{\rho -1}\right]. \end{array}$$

(2) Downward Transition Probability $T_{w,w-1}$

A decrement of the relay buffer occurs when node S successfully forwards a stored relay packet to its destination. The relay buffer at state *w* may consist of packets destined for multiple destinations. Let *v* denote the number of non-empty relay queues among the N−2 possible destinations. We condition on *v* and average over all possible configurations. Given *w* packets in the relay buffer, the total number of possible packet-destination allocations is computed as N−3+ww. Among them, the number of allocations where packets are destined for exactly *v* distinct destinations is calculated as N−2v·(v−1)+(w−v)w−v. Under the uniform mobility model, all allocations are equally likely. Therefore, the conditional probability that exactly *v* relay queues are non-empty is determined as

(7) Pv|w=N−2vw−1w−vN−3+ww.

When node S performs an R–D operation with probability $p_{rd}$, it probes a destination uniformly at random from the N−2 candidate nodes. Conditioned on the fact that *v* relay queues are non-empty, the probability that a single probing attempt results in a successful forwarding is $\frac{v\;p_{rd}}{N-2}$. Accordingly, the probability that the packet is successfully forwarded in the *t*-th probing attempt is ${\left(1-\frac{v\cdot p_{rd}}{N-2}\right)}^{t-1}\frac{v\cdot p_{rd}}{N-2}$. Thus, the overall service probability $\mu_{R}^{v}$ of the relay buffer under the condition that *v* relay queues are non-empty is given by

(8) $$\mu_{R}^{v}=\sum_{t=1}^{\rho }{\left(1-\frac{v\;p_{rd}}{N-2}\right)}^{t-1}\frac{v\;p_{rd}}{N-2}=1-{\left(1-\frac{v\;p_{rd}}{N-2}\right)}^{\rho }.$$

Taking expectation with respect to the conditional probability $P_{v|w}$, the downward transition probability is obtained as

(9) Tw,w−1=∑v=1wPv|w·μRv=∑v=1wN−2vw−1w−vN−3+ww1−1−vprdN−2ρ.

(3) Self-Transition Probability $T_{w,w}$

The remaining transition probability follows from normalization, that is,

(10) $$T_{w,w}=1-T_{w,w+1}-T_{w,w-1}.$$

Based on the above transition characterizations, the relay buffer occupancy process {R(t)} can be modeled as a finite-state Markov chain with a tridiagonal transition structure. The corresponding one-step transition probability matrix T is expressed as

(11) $$T=\left[\begin{array}{cccc} T_{0,0} & T_{0,1} & \; & \\ T_{1,0} & T_{1,1} & T_{1,2} & \\ \; & \ddots & \ddots & \ddots \\ \; & \; & T_{B_{R},B_{R}-1} & T_{B_{R},B_{R}} \end{array}\right].$$

Since all states communicate and the state space is finite, the Markov chain is irreducible and aperiodic, hence ergodic. Therefore, a unique stationary distribution exists.

Let $\psi_{w}$ denote the steady-state probability that *w* packets occupy the relay buffer. Define the stationary distribution vector $\Psi_{\lim}=\left[\psi_{0},\psi_{1},\ldots ,\psi_{B_{R}}\right]$. The stationary probabilities satisfy the global balance equations:

(12) $$\begin{array}{cc} \Psi_{\lim}T & =\Psi_{\lim}, \end{array}$$

(13) $$\begin{array}{cc} \Psi_{\lim}1 & =1, \end{array}$$

where 1 is an all-one column vector of dimension $(B_{R}+1)\times 1$. Solving (12) and (13) yields the steady-state distribution $\Psi_{\lim}$. Note that, due to the finite relay buffer size $B_{R}$, the underlying Markov chain has a finite state space and is thus positive recurrent, ensuring the existence of a unique steady-state distribution regardless of the arrival rate λ and service probability. In particular, the full-buffer probability is given by

(14) $$p_{f}=\psi_{B_{R}}.$$

It is worth noting that the transition matrix T depends on $p_{f}$, while $p_{f}$ itself is determined by the stationary distribution. Consequently, (11)–(14) jointly define a nonlinear fixed-point equation in $p_{f}$. To resolve this coupling, we employ a standard fixed-point iteration procedure [35], as outlined in Algorithm 2. Starting from an initial guess of $p_{f}$, the transition matrix is constructed, the stationary distribution is computed, and the updated value of $p_{f}$ is obtained from $\psi_{B_{R}}$. The iteration continues until convergence, yielding the unique fixed point and thus the exact steady-state distribution of relay buffer occupancy.
**Algorithm 2** Fixed Point Iteration**Input:**   Basic network parameters $\left\{N,M,B_{S},B_{R},\lambda ,\alpha ,\rho \right\}$;**Output:**   Critical value $p_{f}$ (probability that a relay buffer is full);  1:Calculate $p_{sd}$, $p_{sr}$, and $p_{rd}$ according to Equations (1)–(3);  2:Calculate $\mu_{S}=p_{sd}+p_{sr}$, $\tau =\frac{\lambda (1-\mu_{S})}{\mu_{S}\left(1-\lambda \right)}$, and $\phi_{0}=\frac{\mu_{S}-\lambda }{\mu_{S}-\lambda \tau^{B_{S}}}$;  3:Set $x_{1}=0.1$, i=1, and δ a small positive value;  4:**while** ($x_{i}-x_{i-1}\geq \delta )\vee \left(i=1\right)$ **do**  5:    i=i+1;  6:    Use $x_{i-1}$ to replace $p_{f}$ and calculate T according to Equations (6), (9), and (10);  7:    Obtain $\Psi_{\lim}$ by solving Equations (12) and (13);  8:    $x_{i}=\psi_{B_{R}}$;  9:**end while**10:$p_{f}=x_{i}$;11:**return **$p_{f}$;

## 5.3. Fundamental Performance Metrics Derivation

### 5.3.1. Per-Flow Throughput

Recall that the service probability of the source queue in each slot is $\mu_{S}=p_{sd}+p_{sr}$ and the effective packet departure rate from the source queue is $\lambda_{p}=\mu_{S}\left(1-\phi_{0}\right)$. Once a packet leaves the source queue, it is either delivered directly to the destination through an S–D operation or forwarded to a relay via an S–R operation. The fractions of these two events among all service events are $\frac{p_{sd}}{\mu_{S}}$ and $\frac{p_{sr}}{\mu_{S}}$, respectively. Therefore, the throughput contributed by direct transmissions is determined as

(15) $$\Gamma_{S–D}=\lambda_{p}\frac{p_{sd}}{\mu_{S}}=p_{sd}\left(1-\phi_{0}\right).$$

For relay-assisted delivery, successful packet injection into a relay buffer depends on the outcome of the multi-probing procedure. Given that the relay buffer is full with steady-state probability $\psi_{B_{R}}$, the probability that all ρ probing attempts fail equals $\psi_{B_{R}}^{\rho }$. Hence, the probability that at least one probing attempt succeeds is $1-\psi_{B_{R}}^{\rho }$. Accordingly, the throughput achieved via two-hop relaying can be calculated as

(16) $$\Gamma_{S–R–D}=\lambda_{p}\frac{p_{sr}}{\mu_{S}}\left(1-\psi_{B_{R}}^{\rho }\right)=p_{sr}\left(1-\phi_{0}\right)\left(1-\psi_{B_{R}}^{\rho }\right).$$

Combining the above two components, the per-flow throughput is obtained as

(17) $$\Gamma =p_{sd}\left(1-\phi_{0}\right)+p_{sr}\left(1-\phi_{0}\right)\left(1-\psi_{B_{R}}^{\rho }\right).$$

### 5.3.2. Expected E2E Delay

We decompose the E2E delay into two stages:

(18) $$\Theta =\Theta_{S}+\Theta_{D},$$

where $\Theta_{S}$ denotes the queueing delay in the source queue, and $\Theta_{D}$ represents the delivery delay experienced after the packet becomes the HoL packet in the source queue.

Let ${\overset{´}{\phi }}_{k}$ ($0\leq k\leq B_{S}-1$) denote the probability that there are *k* packets in the source queue conditioned on the source buffer not being full. According to [34], ${\overset{´}{\phi }}_{k}$ can be expressed as

(19) $${\overset{´}{\phi }}_{k}=\frac{\lambda }{{\left(1-\lambda \right)}^{2}}\tau^{k}\cdot H_{1}^{-1},\;0\leq k\leq B_{S}-1$$

where $H_{1}$ is the normalization constant. After normalization over the non-full states, the conditional distribution is rewritten as

(20) $${\overset{´}{\phi }}_{k}=\frac{1-\tau }{1-\tau^{B_{S}}}\tau^{k},\;0\leq k\leq B_{S}-1.$$

Then, conditioned on the source buffer not being full, the expected source queue length, denoted as ${\overset{´}{L}}_{S}$, is given by

(21) $${\overset{´}{L}}_{S}=\sum_{k=0}^{B_{S}-1}k{\overset{´}{\phi }}_{k}=\frac{\tau -B_{S}\tau^{B_{S}}+\left(B_{S}-1\right)\tau^{B_{S}+1}}{\left(1-\tau \right)\left(1-\tau^{B_{S}}\right)}.$$

Since the source queue is served with probability $\mu_{S}$ in each slot, the expected source queueing delay is determined as

(22) $$E\left\{\Theta_{S}\right\}=\frac{{\overset{´}{L}}_{S}}{\mu_{S}}.$$

After becoming the HoL packet, the tagged packet waits for a service opportunity, with a mean duration of $1/\mu_{S}$. Once served, the packet is delivered either directly or through relaying. The probabilities of these two cases are proportional to $\Gamma_{S–D}$ and $\Gamma_{S–R–D}$, respectively. Hence, the expected delivery delay can be expressed as

(23) $$E\left\{\Theta_{D}\right\}=\frac{1}{\mu_{S}}+\frac{\Gamma_{S–D}}{\Gamma_{S–D}+\Gamma_{S–R–D}}\cdot 0+\frac{\Gamma_{S–R–D}}{\Gamma_{S–D}+\Gamma_{S–R–D}}\cdot E\left\{\Theta_{R}\right\},$$

where $\Theta_{R}$ denotes the additional delay incurred in the relaying stage.

Let ${\overset{´}{L}}_{R}$ denote the expected number of packets in the relay buffer conditioned on it not being full, which is given by

(24) $${\overset{´}{L}}_{R}=\frac{1}{1-\psi_{B_{R}}}\sum_{w=0}^{B_{R}-1}w\psi_{w}.$$

Owing to the symmetry of the N−2 relay queues in the relay buffer, the mean number of packets in a single relay queue is ${\overset{´}{L}}_{R}/\left(N-2\right)$. Under the MP-2HROR scheme, the service probability of a single relay queue in one slot is determined as

(25) $$\mu_{r}=1-{\left(1-\frac{p_{rd}}{N-2}\right)}^{\rho }.$$

Thus, the expected delay experienced at the relaying stage is calculated as

(26) $$E\left\{\Theta_{R}\right\}=\frac{\frac{{\overset{´}{L}}_{R}}{N-2}+1}{\mu_{r}}=\frac{N-2+{\overset{´}{L}}_{R}}{\left(N-2\right)\left[1-{\left(1-\frac{p_{rd}}{N-2}\right)}^{\rho }\right]}.$$

Integrating the above analytical results yields the final expression for the expected E2E delay:

(27) $$\begin{array}{cc} E\{\Theta \} & =\frac{1-\left(B_{S}+1\right)\tau^{B_{S}}+B_{S}\tau^{B_{S}+1}}{\left(1-\tau \right)\left(1-\tau^{B_{S}}\right)\mu_{S}}+\frac{p_{sr}\left[\left(N-2\right)\left(1-\psi_{B_{R}}^{\rho }\right)+\sum_{z=0}^{\rho -1}\psi_{B_{R}}^{z}\cdot \sum_{w=0}^{B_{R}-1}w\psi_{w}\right]}{\left(N-2\right)\left[p_{sd}+p_{sr}\left(1-\psi_{B_{R}}^{\rho }\right)\right]\left[1-{\left(1-\frac{p_{rd}}{N-2}\right)}^{\rho }\right]}. \end{array}$$

## 6. Simulation Results

In this section, we first conduct simulations to verify the efficiency of our theoretical performance evaluation for the buffer-constrained WSN with the multi-probing opportunistic routing scheme. Then, we explore how key system parameters influence network performance through extensive numerical experiments.

### 6.1. Simulation Settings

To validate the analytical results, we built a dedicated event-driven simulator in C++ that faithfully emulates node mobility, stochastic packet arrivals, buffer evolution, channel access contention, and packet forwarding operations under the MP-2HROR scheme. In particular, the special case ρ=1 corresponds to the conventional two-hop relaying scheme without multi-probing, which is adopted as a baseline for performance comparison throughout the simulations. Each simulation run spans for $2\times 10^{8}$ time slots to ensure statistical reliability. To eliminate transient effects, only performance samples collected during the last 80% of the simulation horizon are used for evaluation, after the system dynamics have stabilized.

### 6.2. Validation

**Figure 4:**
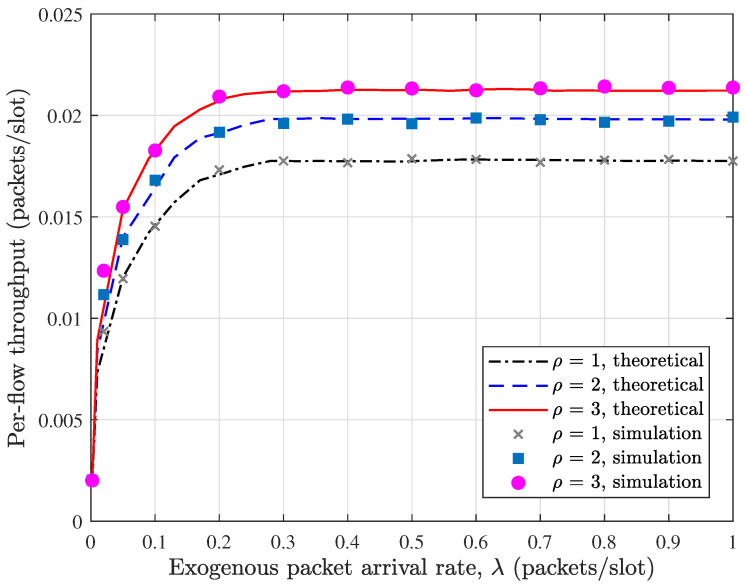
Validation of per-flow throughput in buffer-constrained WSN with MP-2HROR scheme. N=72, M=6, B=10, $B_{S}=B_{R}=5$, α=0.5.

In Figure 4, we compare the theoretical evaluation with simulation results for the per-flow throughput in the buffer-constrained WSN under the MP-2HROR scheme, where N=72, M=6, $B_{S}=B_{R}=5$, and α=0.5. Across the entire range of the exogenous arrival rate λ, the theoretical curves almost coincide with the corresponding simulation results, demonstrating the tightness of the proposed performance evaluation framework. The consistency holds not only in the low-load region but also near saturation, confirming that the derived steady-state distributions accurately capture the buffer dynamics and medium access behaviors. It can be observed from Figure 4 that the per-flow throughput increases rapidly when λ is small, as the system operates in an underloaded regime where most packets can be successfully delivered. As λ continues to grow, the throughput gradually approaches its upper bound, reflecting the intrinsic service capability of the network under the given configuration. This saturation phenomenon indicates that the throughput becomes constrained by the forwarding opportunities rather than the input rate. Moreover, increasing the probing depth ρ consistently enlarges the achievable throughput, and the performance gap becomes more pronounced in the high-load region. This confirms that multi-probing effectively mitigates transmission opportunity loss caused by buffer blocking and enhances channel utilization efficiency.

**Figure 5:**
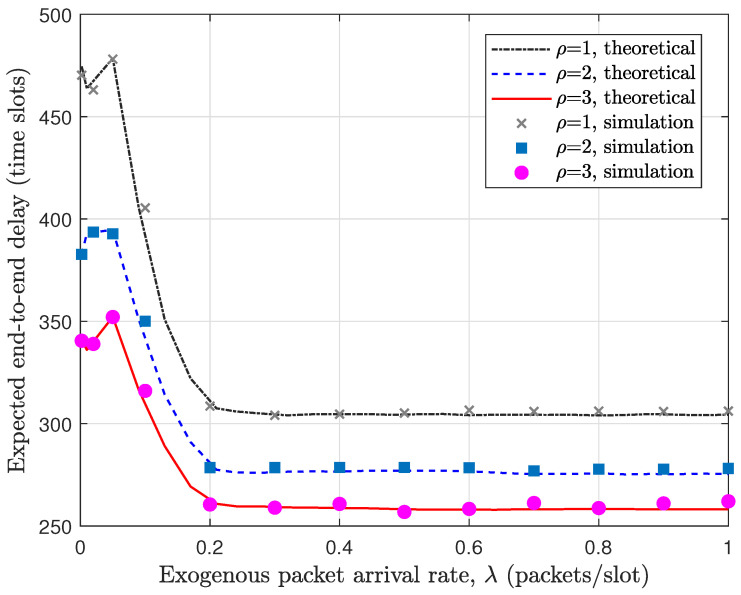
Validation of expected E2E delay in buffer-constrained WSN with MP-2HROR scheme. N=72, M=6, B=10, $B_{S}=B_{R}=5$, α=0.5.

Figure 5 illustrates the corresponding results for the expected E2E delay under the same parameter setting. We can see that the simulation results closely track the theoretical curves, validating the accuracy of the delay modeling. An interesting phenomenon is that the expected delay decreases as λ increases in the high-load regime. This behavior stems from the finite buffer constraint: when λ is large, packets are more likely to be dropped due to buffer overflow, and only successfully delivered packets are included in the delay statistics. Consequently, the average delay is dominated by packets that experience relatively shorter waiting times. In addition, increasing ρ significantly reduces the expected delay across all traffic loads. This improvement is particularly evident in the moderate-load regime, where additional probing opportunities substantially increase the probability of successful forwarding within a time slot, thereby shortening queueing time at both source and relay nodes. Overall, the delay results further verify that multi-probing enhances both service efficiency and timeliness in the buffer-constrained WSN.

### 6.3. Performance Discussions

**Figure 6:**
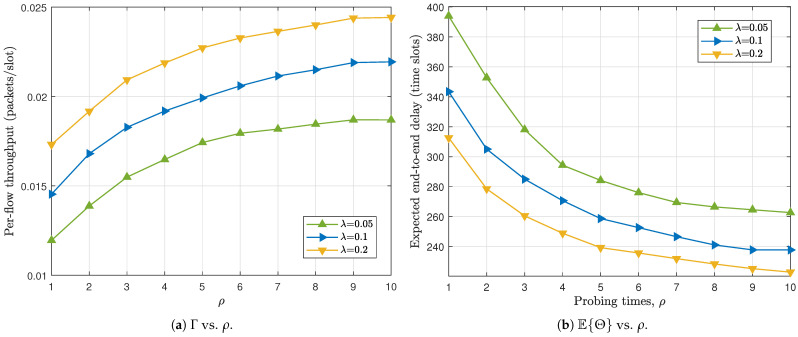
Impact of probing times on the network performance. N=72, M=6, $B_{S}=B_{R}=5$, α=0.5.

We investigate in Figure 6 how the probing depth ρ influences the network performance of the buffer-constrained WSN employing the MP-2HROR scheme, where we set N=72, M=6, $B_{S}=B_{R}=5$, α=0.5, and λ∈{0.05,0.1,0.2}. It can be observed from Figure 6a that increasing ρ consistently improves the throughput under all traffic loads. This improvement stems from the enlarged opportunity provided by multi-probing, i.e., when more relay candidates can be sequentially examined after channel access, the probability of identifying an available relay with a non-full buffer increases, thereby enhancing the effective packet injection rate into the network. Figure 6b shows a clear decreasing trend of the expected E2E delay with respect to ρ for all cases. With deeper probing, packets residing in relay queues have a higher likelihood of being forwarded during each R–D operation, which accelerates packet departure from the network and reduces the average residence time. In addition, the delay reduction effect is particularly significant when ρ increases from small values (e.g., ρ=1 to ρ=3), indicating that shallow probing severely limits forwarding opportunities. It is also worth noting that both throughput improvement and delay reduction exhibit diminishing returns as ρ becomes large. Beyond a certain probing depth, the marginal performance gain becomes negligible. This phenomenon reflects the tradeoff between probing diversity and practical overhead.

From a system-level perspective, the probing depth ρ plays a critical role in jointly shaping transmission efficiency, energy consumption, and traffic dynamics. Increasing ρ improves transmission opportunity utilization and reduces delay by enabling more flexible relay selection. However, it also incurs additional signaling and probing overhead, leading to higher energy consumption. Moreover, a larger ρ accelerates packet forwarding and injection processes, which can help mitigate congestion under moderate traffic load, but may also increase buffer pressure and packet dropping under heavy load. Therefore, the choice of ρ should balance performance gains, energy efficiency, and traffic management requirements in practical WSN deployments.

**Figure 7:**
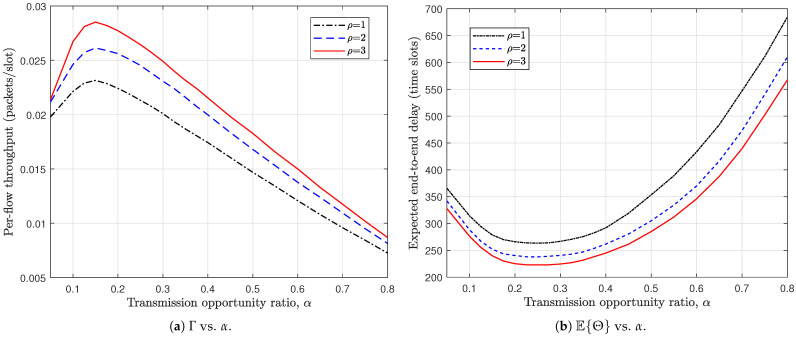
Impact of transmission opportunity ratio on the network performance. N=72, M=6, $B_{S}=B_{R}=5$, λ=0.1.

We further investigate the impact of the transmission opportunity ratio α on the network performance. From Figure 7a, it can be observed that the per-flow throughput exhibits a unimodal behavior with respect to α. Specifically, as α increases from a small value, Γ first improves, reaches a peak, and then gradually declines. This phenomenon reflects the intrinsic balance between packet injection (S–R operation) and packet delivery (R–D operation). When α is too small, insufficient S–R transmissions limit the rate at which new packets enter the relay nodes, resulting in under-utilization of the network resources. Conversely, when α becomes large, excessive S–R operations lead to congestion in relay buffers and insufficient R–D opportunities for packet forwarding, which in turn degrades the effective throughput. Therefore, there exists an optimal α that properly balances the two types of operations. A similar tradeoff is observed in Figure 7b for the expected E2E delay. The delay curve also demonstrates a convex shape, decreasing initially and then increasing as α grows. When α is small, packets experience longer waiting times in the source queues due to limited injection opportunities. As α increases to a moderate level, the system achieves a better coordination between packet dissemination and delivery, thereby shortening the overall delay. However, further increasing α shifts too many transmission opportunities toward S–R operations, causing packet accumulation in relay buffers and prolonging the E2E delay. An interesting observation is that the optimal α, either maximizing throughput or minimizing delay, is consistently smaller than 0.5 for all considered probing depths ρ. This indicates that allocating relatively more transmission opportunities to R–D operations is beneficial for overall performance. Intuitively, prioritizing packet delivery helps maintain a healthy relay layer and prevents buffer congestion, which in turn sustains both high throughput and low delay. This result highlights the importance of properly designing the transmission opportunity allocation rather than adopting a symmetric configuration.

**Figure 8:**
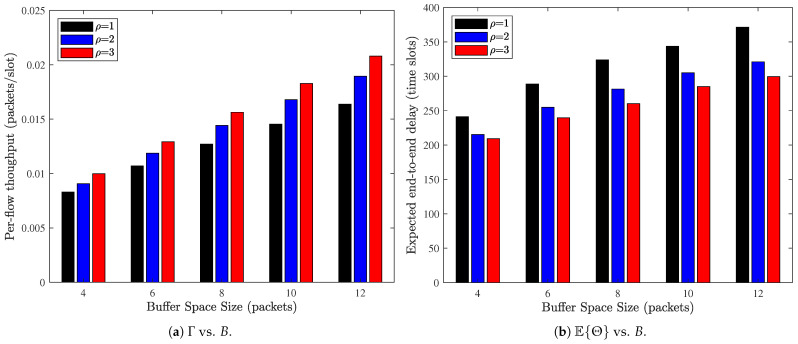
Impact of buffer space size on the network performance. N=72, M=6, $B_{S}=B_{R}=\frac{1}{2}B$, λ=0.1, α=0.5.

Figure 8 illustrates the impact of the total buffer space size *B* on the network performance, where the source and relay buffers are symmetrically configured as $B_{S}=B_{R}=B/2$. From Figure 8a, it is evident that the per-flow throughput increases steadily as the buffer space size *B* grows for all considered probing times ρ. Enlarging the buffer capacity effectively alleviates packet drops caused by buffer overflow at both the source and relay nodes. As a result, a larger fraction of generated packets can successfully enter and traverse the network, leading to improved throughput. Moreover, the performance gain brought by increasing ρ remains consistent across different buffer sizes, indicating that the benefit of multi-probing and that of larger buffer capacity are complementary rather than substitutive. In contrast, Figure 8b shows that the expected E2E delay increases with *B*. Although a larger buffer reduces packet loss, it also allows more packets to be stored and queued in the system, thereby increasing the average queue length at both the source and relay nodes. Consequently, packets tend to experience longer waiting times before being forwarded, which leads to a higher E2E delay. This result reveals a fundamental tradeoff, i.e., expanding the buffer capacity improves reliability and throughput at the expense of increased latency. We can understand that while multi-probing accelerates packet forwarding, it cannot completely offset the delay growth induced by larger queueing spaces. Therefore, buffer provisioning and probing configuration should be jointly optimized to achieve a desirable balance between throughput and delay performance.

**Figure 9:**
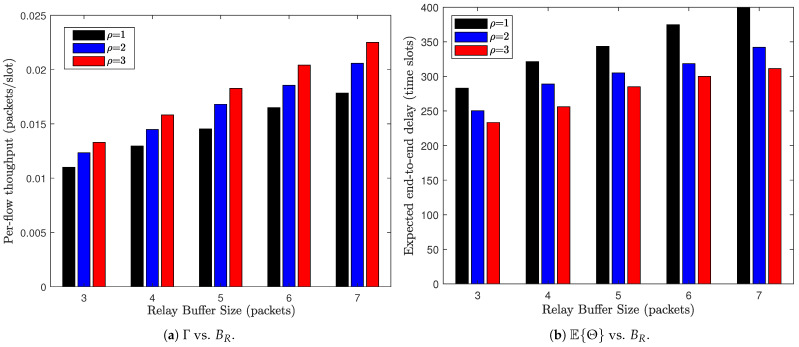
Impact of buffer space allocation on the network performance. N=72, M=6, B=10, λ=0.1, α=0.5.

We further summarize in Figure 9 the impact of buffer space allocation under a fixed total buffer size B=10, where the relay buffer size $B_{R}$ varies, and the source buffer size is correspondingly adjusted as $B_{S}=B-B_{R}$. From Figure 9a, we observe that the per-flow throughput increases monotonically as $B_{R}$ grows. Allocating more space to the relay buffer enables relay nodes to accommodate more in-transit packets, thereby reducing relay-side blocking and enhancing the probability that S–R transmissions are successfully admitted into the network. On the other hand, Figure 9b shows that the expected E2E delay increases with $B_{R}$. While enlarging the relay buffer mitigates packet loss, it simultaneously expands the relay queueing space, allowing packets to reside longer within the network before reaching their destinations. More importantly, Figure 9 reveals a structural tradeoff induced by buffer partitioning. Under a fixed total buffer budget, shifting storage capacity from the source buffer to the relay buffer prioritizes network-wide packet carrying capability over local packet retention. In other words, nodes that allocate more buffer space for relaying (i.e., behaving more “cooperatively”) enhance the overall throughput of the WSN, but at the cost of increased delay due to longer relay queues. Therefore, optimal buffer partitioning should carefully balance throughput maximization and delay control, depending on the application requirements (e.g., delay-sensitive vs. throughput-oriented scenarios).

**Figure 10:**
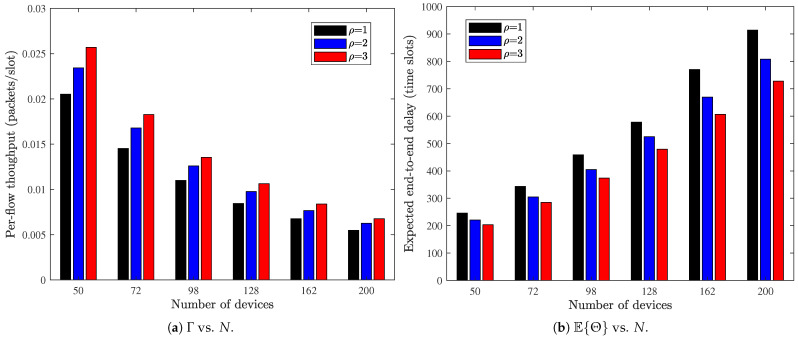
Impact of number of devices on the network performance. $M=\sqrt{N/2}$, $B_{S}=B_{R}=5$, λ=0.1, α=0.5.

Finally, we examine the scalability of the MP-2HROR scheme by varying the network size *N* while keeping the node density constant, i.e., $N/M^{2}=2$. We can observe from Figure 10 that as the network scale increases (i.e., *N* increases), both throughput and delay performance deteriorate. This observation suggests that although purely protocol-level enhancements (e.g., increasing probing times) can mitigate the performance degradation to some extent, they cannot change the degradation scaling law. To sustain performance in large-scale deployments, it is necessary to reinforce the physical or resource configuration of devices, such as enlarging the buffer capacity, so that the per-node service capability scales with network size.

## 7. Conclusions

In this paper, we proposed a multi-probing two-hop relaying opportunistic routing scheme for buffer-constrained decentralized wireless sensor networks and established a unified analytical framework for its performance evaluation. Different from most recent related studies that primarily focus on opportunistic forwarding design or simulation-based performance evaluation, this work explicitly captures the coupled effects of finite buffer capacity, transmission opportunity utilization, and node mobility within a rigorous queueing-theoretic and Markov-chain-based framework. Based on this framework, we derived closed-form expressions for the per-flow throughput and the expected end-to-end delay, and validated the analytical results through extensive simulations. The numerical results reveal several important insights. First, multi-probing significantly improves network throughput and reduces end-to-end delay by increasing the probability of identifying suitable relays and delivery opportunities. Second, such performance gains exhibit diminishing returns as the probing depth increases, indicating a fundamental tradeoff between probing diversity and operational overhead. Third, transmission opportunity allocation and buffer configuration play critical roles in balancing throughput and delay performance. These findings provide both theoretical insights and practical design guidelines for highly dynamic buffer-constrained WSNs.

Future work will extend the proposed framework in several directions. First, more general network scenarios with heterogeneous mobility patterns and traffic models will be considered. Second, we will further investigate adaptive or learning-based probing strategies to improve relay selection under limited network state information. In addition, more comprehensive performance comparisons with other routing schemes applicable to highly dynamic WSNs will be conducted to further validate the effectiveness of the proposed approach. Finally, incorporating practical constraints such as energy limitations and imperfect control signaling is also an important direction for future research.

## Data Availability

The source code utilized in this study is available upon reasonable request from the corresponding author.
